# The Image-to-Physical Liver Registration Sparse Data Challenge: comparison of state-of-the-art using a common dataset

**DOI:** 10.1117/1.JMI.11.1.015001

**Published:** 2024-01-08

**Authors:** Jon S. Heiselman, Jarrod A. Collins, Morgan J. Ringel, T. Peter Kingham, William R. Jarnagin, Michael I. Miga

**Affiliations:** a Vanderbilt University, Department of Biomedical Engineering, Nashville, Tennessee, United States; b Memorial Sloan Kettering Cancer Center, Department of Surgery, Hepatopancreatobiliary Unit, New York, New York, United States

**Keywords:** image guidance, registration, sparse data, liver, challenge, accuracy

## Abstract

**Purpose:**

Computational methods for image-to-physical registration during surgical guidance frequently rely on sparse point clouds obtained over a limited region of the organ surface. However, soft tissue deformations complicate the ability to accurately infer anatomical alignments from sparse descriptors of the organ surface. The Image-to-Physical Liver Registration Sparse Data Challenge introduced at SPIE Medical Imaging 2019 seeks to characterize the performance of sparse data registration methods on a common dataset to benchmark and identify effective tactics and limitations that will continue to inform the evolution of image-to-physical registration algorithms.

**Approach:**

Three rigid and five deformable registration methods were contributed to the challenge. The deformable approaches consisted of two deep learning and three biomechanical boundary condition reconstruction methods. These algorithms were compared on a common dataset of 112 registration scenarios derived from a tissue-mimicking phantom with 159 subsurface validation targets. Target registration errors (TRE) were evaluated under varying conditions of data extent, target location, and measurement noise. Jacobian determinants and strain magnitudes were compared to assess displacement field consistency.

**Results:**

Rigid registration algorithms produced significant differences in TRE ranging from 3.8±2.4  mm to 7.7±4.5  mm, depending on the choice of technique. Two biomechanical methods yielded TRE of 3.1±1.8  mm and 3.3±1.9  mm, which outperformed optimal rigid registration of targets. These methods demonstrated good performance under varying degrees of surface data coverage and across all anatomical segments of the liver. Deep learning methods exhibited TRE ranging from 4.3±3.3  mm to 7.6±5.3  mm but are likely to improve with continued development. TRE was weakly correlated among methods, with greatest agreement and field consistency observed among the biomechanical approaches.

**Conclusions:**

The choice of registration algorithm significantly impacts registration accuracy and variability of deformation fields. Among current sparse data driven image-to-physical registration algorithms, biomechanical simulations that incorporate task-specific insight into boundary conditions seem to offer best performance.

## 1 Introduction

Image-to-physical registration is an essential component of surgical navigation systems wherein patient-specific information from preoperative imaging is aligned to the intraprocedural coordinate space of the patient. This alignment process requires an intraoperative data collection step to acquire intraprocedural shape descriptors of the organ of interest. Several methods have been developed for three-dimensional measurement of intraoperative organ geometry, including optical or electromagnetic tracking of tool tips, stereo camera reconstruction, laser range scanning, tracked ultrasound, and cone-beam computed tomography, among others.^1^ However, constraints within the surgical environment require that organ data be collected in an expedient manner compatible with existing surgical workflows. In consideration of these constraints, the intraoperative data most often available to navigation systems consists of intraoperative point clouds collected over a limited extent of the organ surface exposed during the procedure. In soft tissue organs, these intraoperative surface measurements not only drive rigid spatial alignments between preoperative and intraoperative coordinate frames, but they also encode changes in organ shape that occur between preoperative imaging and intraoperative organ presentation. The ability to accurately estimate a complete soft tissue deformation field throughout the organ from these sparse organ surface measurements is a vital objective for many guidance systems involving soft tissue organs. This inference task is the basis of the sparse data challenge herein, which focuses on the application of image-guided liver surgery in which organ deformations frequently reach several centimeters in magnitude.^2^^,^^3^ Rigid and nonrigid registration approaches have been reported in the literature to accomplish image-to-physical alignment, yet direct comparisons of accuracy associated with these methods have long remained out of reach due to a lack of shared validation datasets. Opportunities to compare performance on shared challenge data will provide new insights toward effective common strategies that may be synthesized into the next generation of novel registration algorithms.

At SPIE Medical Imaging 2019, Brewer et al. introduced the first image-to-physical liver registration sparse data challenge^4^ to allow research groups to validate registration algorithms on a shared dataset. This dataset is based on sparse intraoperative data collected from a tissue-mimicking silicone liver phantom under multiple configurations of intraoperative deformation, previously available at Ref. 5. The challenge data consisted of a preoperative liver mesh and 112 intraoperative data patterns sampled from four unique deformed intraoperative poses produced by placing mock surgical packing beneath the posterior surface of the liver phantom. N=159 validation targets were distributed at blinded positions throughout the phantom to allow for an unbiased comparison of ground truth target positions against predicted target positions estimated by any proposed registration algorithm. Each of the 112 data patterns were mapped onto the deformed intraoperative organ poses from real patterns of intraoperative data collected with an optically tracked stylus over the visible patch of the anterior surface during clinical evaluation of an image-guided liver surgery system.^6^^,^^7^ Since the release of the sparse data challenge in 2019, a variety of methods including three rigid, two deep learning, and three biomechanically driven nonrigid registration techniques have been contributed to the challenge. This paper presents results comparing these registration approaches on the common validation dataset and offers the first direct comparison study of sparse data registration accuracy in a phantom simulation of image-guided liver surgery conditions. This paper reports full metrics for target registration errors (TRE) that previously were blinded to participants in the sparse data challenge to ensure impartiality. Furthermore, this work includes a detailed analysis of registration errors across multiple sparse data registration methods on a large common set of scenarios of image-guided navigation in the liver. Deformable registration methods are evaluated for sensitivity to data coverage, variability across anatomical segments, the effect of measurement noise, differences in initial alignment, and biomechanical consistency of the deformation field. Through these analyses, common limitations and effective strategies are identified across a variety of proposed methods to inform future algorithmic development in the domain of image-to-physical sparse data registration algorithms. Finally, the full validation data, including the previously blinded target positions and analysis techniques for the sparse data challenge, will be released hereafter via the Open Science Framework at Ref. 8 as a continuing platform for algorithm characterization and benchmarking.

## 2 Methods

### 2.1 Sparse Data Challenge

The dataset associated with the image-to-physical liver registration sparse data challenge^4^ was generated via a silicone liver phantom fabricated from a mixture of 80% Ecoflex 00-10 platinum-cure silicone, 10% Silicone Thinner, and 10% Slacker Tactile Mutator (Smooth-On Inc., Macungie, Pennsylvania, United States) molded into a patient-specific 3D-printed cast of a CT-segmented liver volume. A total of 159 stainless steel beads were implanted into the silicone phantom as CT-visible validation targets. The liver phantom was removed from its cast, and mock laparotomy pads were placed under the posterior face of the phantom to simulate four different configurations of plausible intraoperative deformations. Repeat CT imaging was performed to establish a baseline phantom configuration representing the undeformed preoperative state and a series of four intraoperative deformation configurations from which the ground truth liver geometry and positions of validation targets were segmented using ITK-SNAP (Kitware Inc., Clifton Park, New York, United States). The 112 registration datasets were constructed by mapping 28 unique sparse data patterns to each of the four deformation states via the data transposition method in Ref. 9. Each sparse data pattern was acquired during a clinical study of an image-guided liver surgery system approved by the institutional review board at Memorial Sloan Kettering Cancer Center and with informed consent of all participants.^6^^,^^7^ The extent of data coverage over the total surface area of the liver varied with each pattern from 20% to 44%, with average extent of 31.7%±6.4%. To simulate intraoperative instrumentation noise, 21 of 28 data patterns (84 datasets) were mapped to the intraoperative liver geometry with sinusoidal noise of 2-mm amplitude, and the remaining 7 data patterns (28 datasets) were mapped to the intraoperative liver surfaces without additional noise. Randomized rotations and translations were applied to each dataset to ensure that the sparse intraoperative point clouds from each dataset were treated independently. Random rotations were sampled uniformly in SO(3) via normalized axis-angle parameters $e_{i}\in [-1,1]$ for ei∈e^ and θ∈[0,2π], whereas translations were randomly sampled using a uniform distribution in the range $t_{i}\in [-100,100]$ mm for $t_{i}\in t$.

**Fig. 1:**
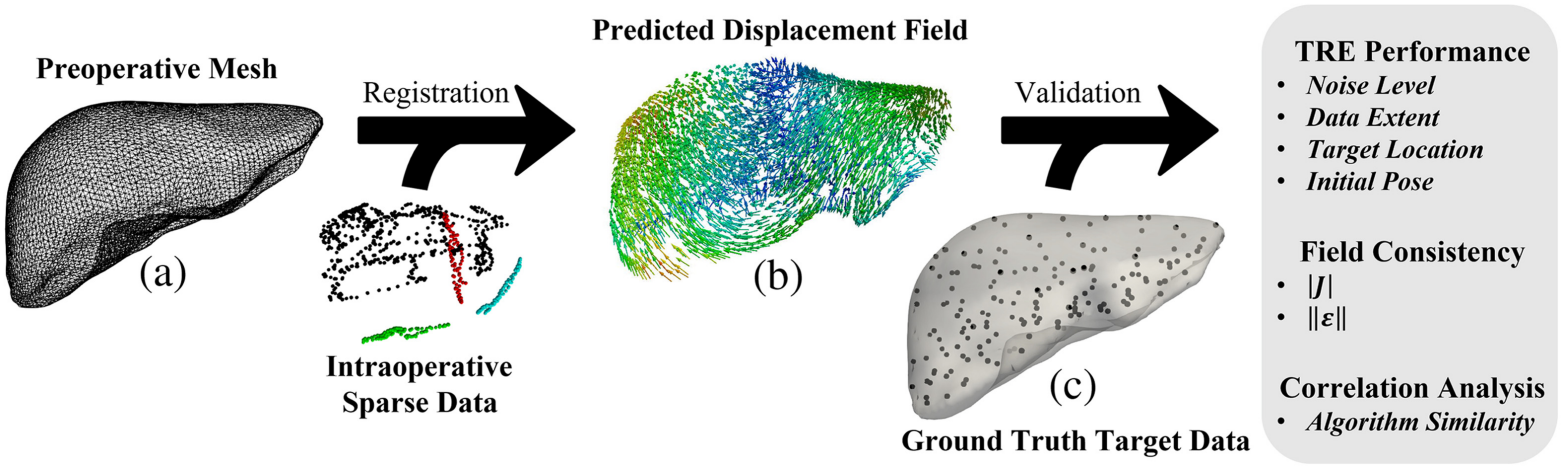
Sparse data challenge registration task. (a) A reference mesh of the non-deformed preoperative liver is provided; it must be registered to 112 patterns of intraoperative sparse data that were collected after one of four unknown deformations were applied to the organ. (b) The registration method generates dense displacement fields for mapping the preoperative liver to the intraoperative data frame (rendered with rigid component removed). (c) Ground truth locations of 159 target locations distributed throughout the liver were previously blinded to participants and serve as validation data. Registration performance was assessed via TRE, which was stratified across variations in clinically relevant factors including measurement noise, data extent, target location, and algorithm initialization. Furthermore, registration performance was compared according to consistency measures of the displacement field, and inter-method similarity was assessed via correlation analysis.

**Fig. 2:**
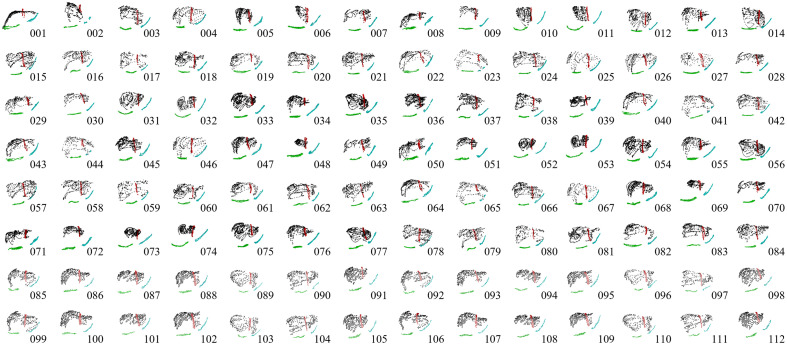
112 patterns of intraoperative surface data provided in the sparse data challenge to drive the image-to-physical registration task. Each intraoperative sparse data pattern was clinically collected during image-guided liver surgery and mapped to the deformed organ phantom. Points associated with the anterior surface of the liver are shown in black, the falciform ligament in red, the right inferior ridge in green, and the left inferior ridge in blue.

Figure 1 illustrates the structure of the sparse data challenge. Given the 112 sparse intraoperative datasets and the initial preoperative liver volume in Fig. 1(a), the task of the challenge is to perform a registration that most accurately predicts the deformed state of the whole organ based on the limited information provided by each sparse data pattern. Registration results are provided according to dense displacement fields [Fig. 1(b)] defined over the preoperative liver volume. Displacements at the blinded validation target locations [Fig. 1(c)] are then interpolated to determine the registration errors of the estimated target positions. The total variation in sparse data patterns across the 112 registration instances is depicted in Fig. 2. Additionally, the sparse data challenge was structured to provide participants with the ability to inform algorithmic development in a restricted manner. An incomplete set of ground truth data for 35 of 159 target positions in 4 of the 112 datasets drawn from 2 of the 4 underlying deformation poses was provided to participants. Furthermore, a web portal was implemented using Amazon Web Services (Amazon Web Services Inc., Seattle, Washington, United States) to allow participants to upload in-progress and finalized registration results. These results were automatically processed to yield coarse summary measures of average TRE across the full dataset and stratified across low, medium, and high ranges of data extent. These results files were hosted on a publicly available dashboard at Ref. 5 for the purposes of benchmarking and to offer a limited capability for hyperparameter characterization and algorithmic tuning among participants. The dataset and contributed results will continue to be available through the Open Science Framework at Ref. 8 upon closure of the challenge site.

### 2.2 Evaluation

The primary endpoint of the sparse data challenge is the set of TRE associated with individual registrations to the 112 unique intraoperative data patterns that comprise the dataset. Registration errors of the 159 validation targets in each registration were measured according to

$${\mathrm{TRE}}_{i}={\left\|x_{i,\mathrm{GT}}-x_{i,\mathrm{est}}\right\|}_{2},$$ (1)

where $x_{i,\mathrm{GT}}$ is the ground truth intraoperative position of target i and $x_{i,\mathrm{est}}$ is the estimated position of target i predicted by the registration. Average TRE ($\bar{\mathrm{TRE}}$) of each registration was computed as the mean of ${\mathrm{TRE}}_{i}$. Further, $\bar{\mathrm{TRE}}$ among registrations to each of the 112 datasets were statistically compared between registration methods via the Friedman test at a significance level of α=0.05 with Bonferroni correction applied to reported p-values to adjust for multiple comparisons.

Furthermore, the sensitivity of TRE to digitization noise was evaluated. The impact of digitization noise was quantified through measures for noise efficiency $E_{N}$ and noise degradation $D_{N}$ according to

$$E_{N}=A_{N}/{\overset{¯}{\mathrm{TRE}}}_{N},$$ (2)

$$D_{N}=({\bar{\mathrm{TRE}}}_{N}-{\bar{\mathrm{TRE}}}_{N0})/A_{N},$$ (3)

for a noise level of $A_{N}=2\;\mathrm{mm}$ and average TRE of noisy and noise-free registrations ${\bar{\mathrm{TRE}}}_{N}$ and ${\overset{¯}{\mathrm{TRE}}}_{N0}$, respectively. The impact of digitization noise on the average value and variance of $\bar{\mathrm{TRE}}$ was assessed for each method using the Wilcoxon rank sum and Brown–Forsythe tests, respectively, at a significance level of α=0.05.

TRE performance was also stratified by the effects of surface data coverage and target location within the segmental anatomy of the liver. The extent of surface data coverage was defined according to the method of Ref. 3, in which the data extent was computed as the percentage of total organ surface area encompassed by an alpha shape fit to the sparse intraoperative point cloud. Moreover, targets were stratified into the eight anatomical Couinaud segments to evaluate the potential impact of clinical designation of lesion location on registration performance. Anatomical segments S2 through S8 each contained 19 to 30 individual targets. The caudate lobe (S1) was not evaluated for lack of a sufficient number of validation targets in this region of the liver. Similarly, the effects of surface data coverage and target segment location on registration accuracy were assessed via the Friedman test with Bonferroni correction with significance level α=0.05.

The impact of initial rigid pose on TRE was also compared among methods that had code available to the authors. Sensitivities to initial alignment were assessed via two-sample Kolmogorov–Smirnov tests at a significance level of α=0.05 to detect whether statistical distributions of $\bar{\mathrm{TRE}}$ associated with the deformable registration methods differed under three conditions of initial rigid alignment: (1) an optimal point-based rigid registration of ground truth target positions, (2) an iterative closest point (ICP) rigid registration algorithm with manual initialization and refinement, and (3) a fully automatic salient feature weighted ICP (wICP) algorithm.

Displacement fields of the deformable registration algorithms were also analyzed for biomechanical consistency by computing the norm of the rotation-invariant Green strain tensor ‖ε‖ and the Jacobian determinant |J| of the displacement fields applied to the liver in each registration, according to

$$\|\varepsilon \|=\frac{1}{2}{\left\|\nabla u^{T}+\nabla u+\nabla u^{T}\nabla u\right\|}_{2},$$ (4)

|J|=|∇u+I|, (5)

for each element displacement gradient tensor ∇u, where ${\left\|\cdot \right\|}_{2}$ is the L2 matrix norm, |·| is the matrix determinant, and I is the identity matrix.

Finally, correlations among the resulting ${\mathrm{TRE}}_{i}$ of each method were compared in a Pearson correlation plot to identify similarities in target error behaviors among registration methods.

## 3 Registration Comparators

Eight liver registration strategies were contributed and evaluated in the sparse data challenge. Complete submissions were made to the sparse data challenge for each method by providing displacement fields for registrations to all 112 sparse data patterns. These methods consisted of the following techniques:

1. An optimal point-based rigid registration (PBR) between ground-truth preoperative and intraoperative target positions as a common comparator to the best rigid alignment.
2. An ICP rigid registration with a manually established initial pose and manual reinitialization when necessary.^10^
3. A fully automatic salient feature wICP rigid registration (Clements).^11^
4. A deep learning method based on organ-data signed distance maps (Pfeiffer).^12^
5. A deep learning method based on probabilistic shape occupancy maps (Jia).^13^
6. A biomechanical finite element method based on linearized iterative boundary condition reconstruction (Heiselman).^14^^,^^15^
7. A biomechanical finite element method based on adjoint boundary condition reconstruction (Mestdagh).^16^
8. A biomechanical reconstructive method based on analytical closed-form regularized Kelvinlet solutions to point load perturbations (Ringel).^17^

Table 1 briefly summarizes the key features of these algorithms, which are described in detail in Sec. 7.

**Table 1:** Algorithmic profile of registration methods contributed to the sparse data challenge.

Registration Method	PBR	ICP	wICP	Pfeiffer^12^	Jia^13^	Heiselman^14^^,^^15^	Mestdagh^16^	Ringel^17^
**Support**	Rigid	Rigid	Rigid	Deformable	Deformable	Deformable	Deformable	Deformable
**Model type**	Classical	Classical	Classical	Deep learning	Deep learning	Classical	Classical	Classical
**Transformation model**	Rigid	Rigid	Rigid	Learned	Biomechanical	Biomechanical	Biomechanical	Biomechanical
(i) **Requires initial rigid alignment?**	✗	✓	✗	✓	✓	✓	✓	✓
(ii) **Refines initial rigid alignment parameters?**	—	✓	—	✗	✓	✓	✗	✓
**Model architecture**	—	—	—	CNN	PCNN	FEM	FEM	Analytic
(i) **Requires training data?**	✗	✗	✗	✓	✓	✗	✗	✗
**Biomechanical simulation**	—	—	—	Hyperelastic (FEM)	Linear elastic (FEM)	Linear elastic (FEM)	Linear elastic (FEM)	Linear elastic (analytic)
(i) **Reconstructs boundary conditions?**	—	—	—	✗	✓	✓	✓	✓
(ii) **Anatomically informed boundary conditions?**	—	—	—	✗	✗	✓	✓	✗
**Model-to-data constraint**	Fiducial correspondence	Closest point distance	Closest point distance	Signed distance fields	Learned	Surface normal distance	Closest point distance	Surface normal distance
**Optimization technique**	—	Iterative	Iterative	—	Levenberg–Marquardt	Levenberg–Marquardt	Quasi-Newton (BFGS)	Levenberg–Marquardt
**Regularization**	None	None	Feature weights	None	Strain energy	Strain energy	None	Strain energy
**Interaction**	—	Manual	Semi-automatic	Automatic	Automatic	Automatic	Automatic	Automatic
**Computation time**	∼5 ms	∼300 ms ^a^	∼350 ms	∼130 ms ^b^	Not reported	∼5 s ^c^	Not reported	∼40 s
**Code availability**	Open source	Open source	Closed source	Open source	Closed source	Closed source	Closed source	Closed source

a Additional intraoperative time required for manual initialization, review, adjustment, and re-registration.

b Inference time only; additional intraoperative time required for construction of distance maps (not reported).

c Reconstruction time only; additional preoperative time required for finite element precomputation (∼20  s with distributed parallelization).

## 4 Results

### 4.1 Target Registration Errors

**Fig. 3:**
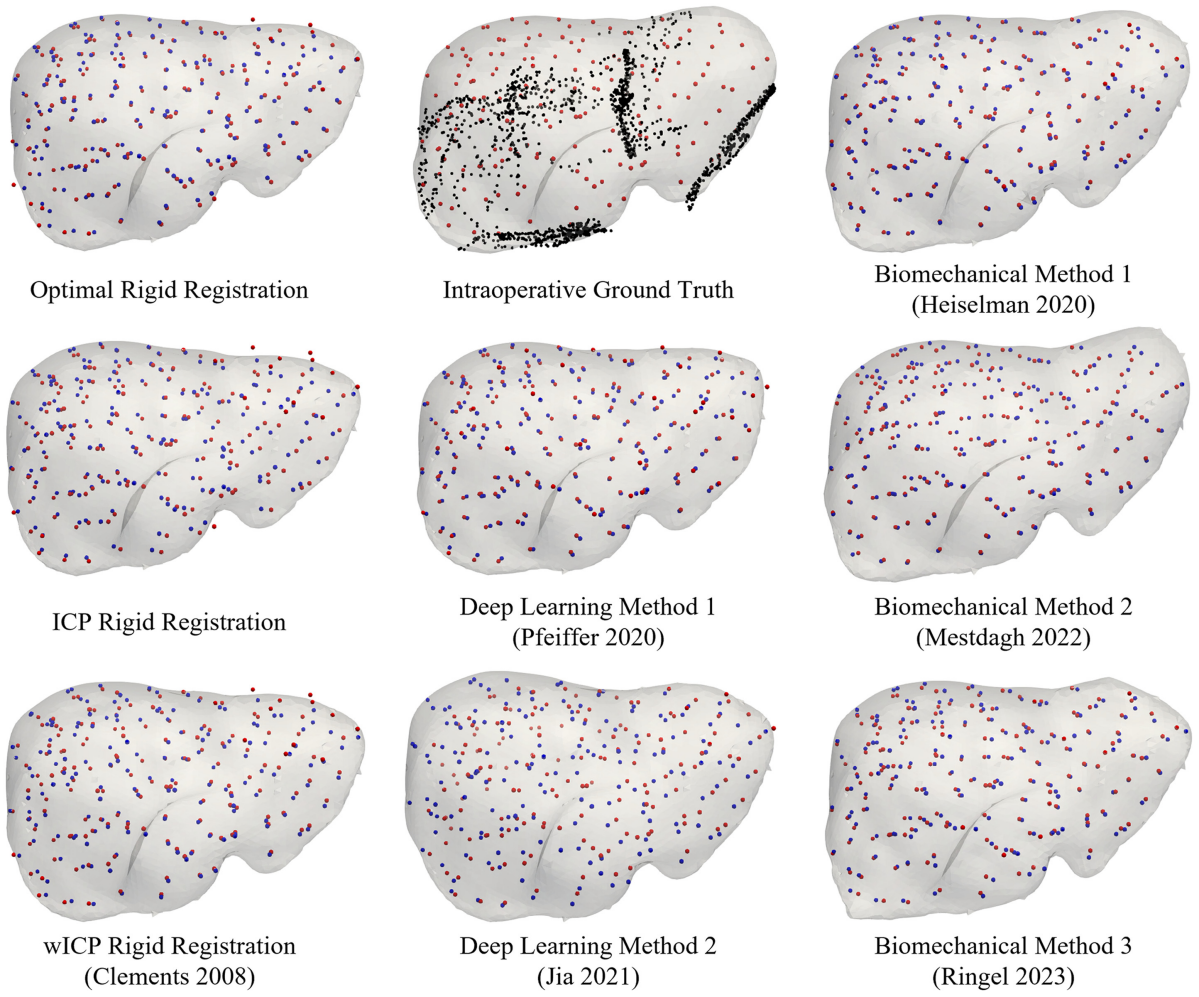
Registration results from the sparse data challenge corresponding to one of the 112 registration scenarios. The top center panel shows the intraoperative ground truth target positions (red) and deformed liver shape (gray), alongside the intraoperative sparse surface data pattern provided for the registration task (black). In all other panels, the resulting target positions predicted by each registration method (blue) are compared against the ground truth target positions (red). The deformed liver shape predicted by each registration method is also shown in gray.

**Fig. 4:**
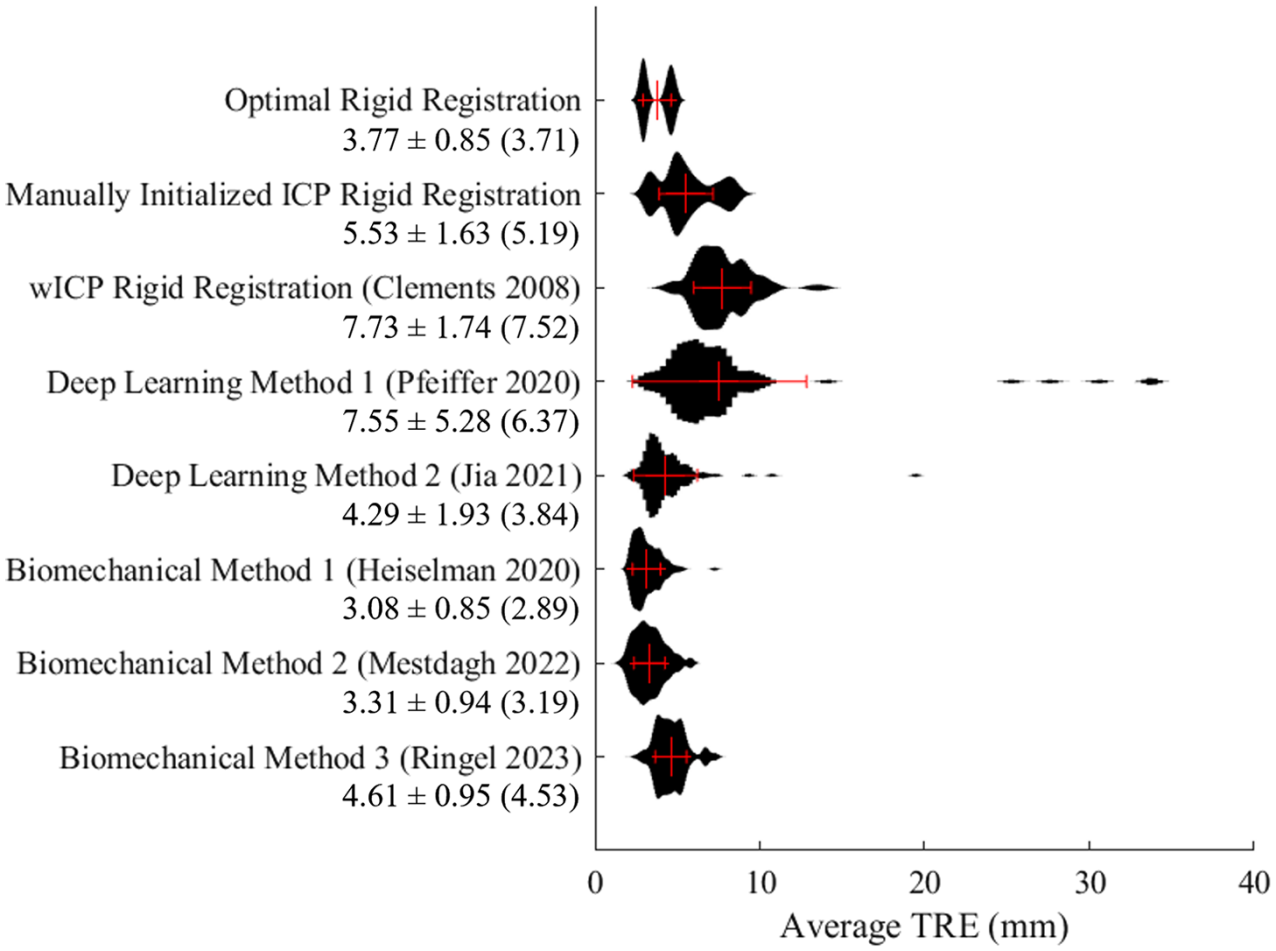
Distributions of average TRE of each of the 112 registration instances in the sparse data challenge. Mean and standard deviation are plotted with red bars. Quantitative measures are reported as mean ± standard deviation (median) in units of mm.

Registration results from one of the 112 sparse datasets are visualized in Fig. 3 for the rigid and deformable registration comparators, with predicted and ground truth target positions displayed alongside the intraoperative distribution of sparse data driving the registration. Figure 4 illustrates the distribution of average TRE within registrations to each of the 112 datasets and summarizes the overall mean, standard deviation, and median performance of each method. Among rigid registration methods, the manually supervised ICP approach led to significantly lower average TRE than the fully automatic salient feature wICP algorithm (p<0.001), although both ICP (p<0.001) and wICP (p<0.001) rigid alignments led to significantly worse average TRE than the optimal PBR alignment of targets. With respect to the deformable registration methods, although the biomechanical boundary condition reconstruction methods of Heiselman and Mestdagh did not significantly differ from each other (p>0.99), the method of Heiselman provided registrations with significantly lower TRE than the optimal point-based rigid registration of targets (p=0.048), whereas the method of Mestdagh did not significantly improve over the optimal PBR (p=0.81). The deep learning method by Jia was not found to produce TRE higher than either the optimal point-based registration (p=0.37) or Mestdagh (p=0.09), but significantly worse performance was detected compared to Heiselman (p<0.001). Meanwhile, the biomechanical method by Ringel produced average TRE that did not significantly differ from Jia (p=0.10) or the ICP method (p=0.39), and the deep learning method by Pfeiffer was significantly less accurate than ICP (p=0.011) but not wICP (p=0.93). All other pairs of registration methods were found to produce significantly different levels of average TRE (all p<0.01).

### 4.2 Effect of Surface Data Coverage

**Fig. 5:**
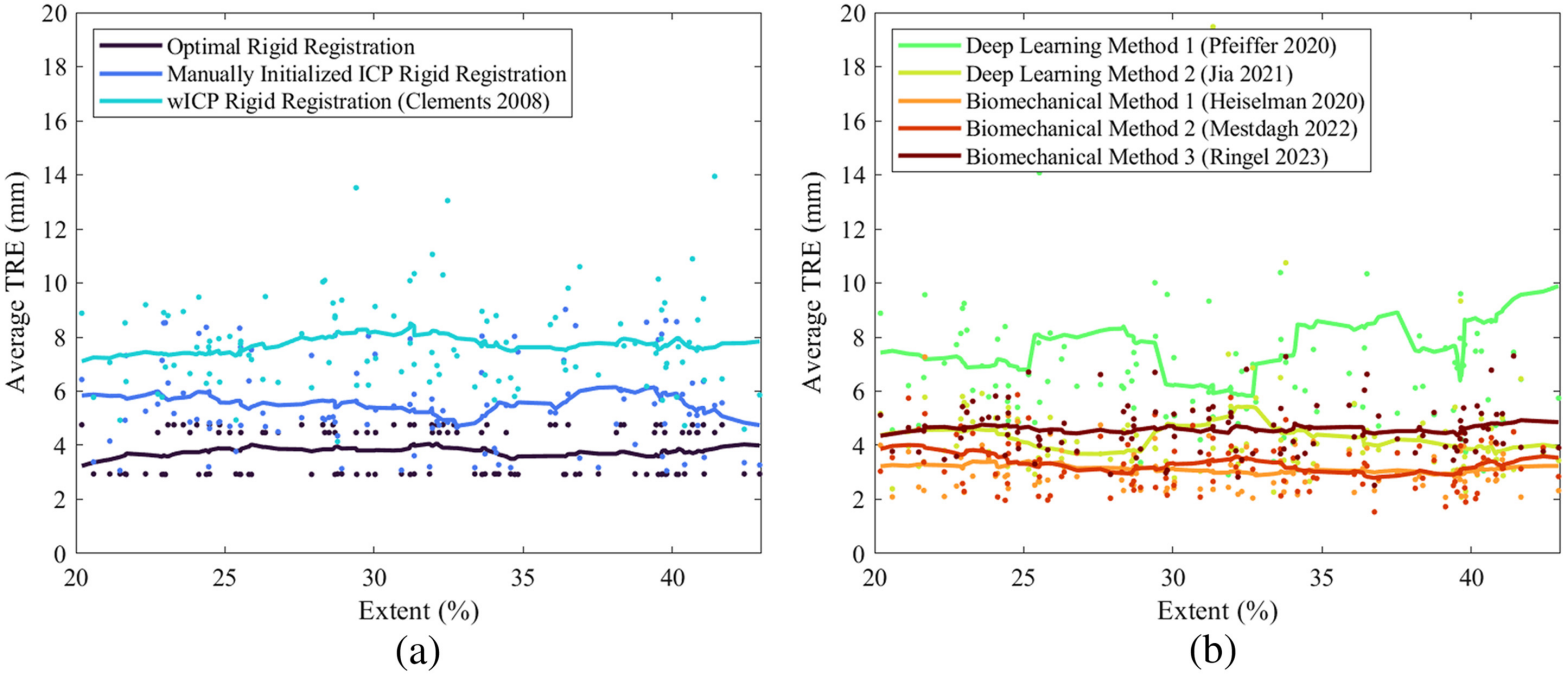
Mean TRE of rigid registration methods (a) and deformable registration methods (b) compared against the extent of sparse surface data coverage on the liver. Solid lines indicate moving average.

The 112 sparse intraoperative datasets were stratified according to the extent of surface data coverage as a percentage of the total liver surface area. Of the 112 registration sets, 35 cases were associated with surface coverage between 20–28% extent, 42 cases between 28–36% extent, and 35 cases between 36–44% extent. Figure 5 plots the average TRE of each registration according to the extent of sparse surface data coverage provided over the liver. Across all registration methods, registration performance did not significantly differ as a function of surface data extent (p=0.88). With respect to the rigid registration algorithms, ICP with manual initialization and refinement achieved average TRE values closer to the optimal rigid registration than the wICP algorithm over the full range of data extent (p<0.001), which suggests that additional bias introduced by wICP feature weighting may lead to suboptimal rigid alignments despite its excellent practical utility in intraoperative workflows. In comparison with the deformable registration algorithms, Fig. 5 qualitatively shows that the finite element-based biomechanical methods 1 and 2 (Heiselman^12^ and Mestdagh^14^) outperform or achieve similar performance to the optimal point-based rigid registration, whereas the deep learning methods are associated with the highest errors among the deformable registration methods. These trends mirror the significance patterns reported in the previous section. The regularized Kelvinlet method (Ringel^15^) performed similarly to the deep learning method by Jia that incorporates a biomechanical simulation workflow, with slightly improved qualitative stability across extent ranges. Both the methods of Ringel and Jia offered significant improvements over wICP-based rigid registration (p<0.001) but failed to outperform the globally optimal rigid point-based registration. The relative stability of average TRE across the low to high extent ranges is consistent with the work of Refs. 3 and 18, which showed that rigid and nonrigid registration methods tend to reach an error floor plateau beyond extent ranges of ∼20%. Yet, it should be noted that the deep learning approaches seem to exhibit less consistency in their performance across variations in surface data coverage.

### 4.3 Effect of Target Location

**Fig. 6:**
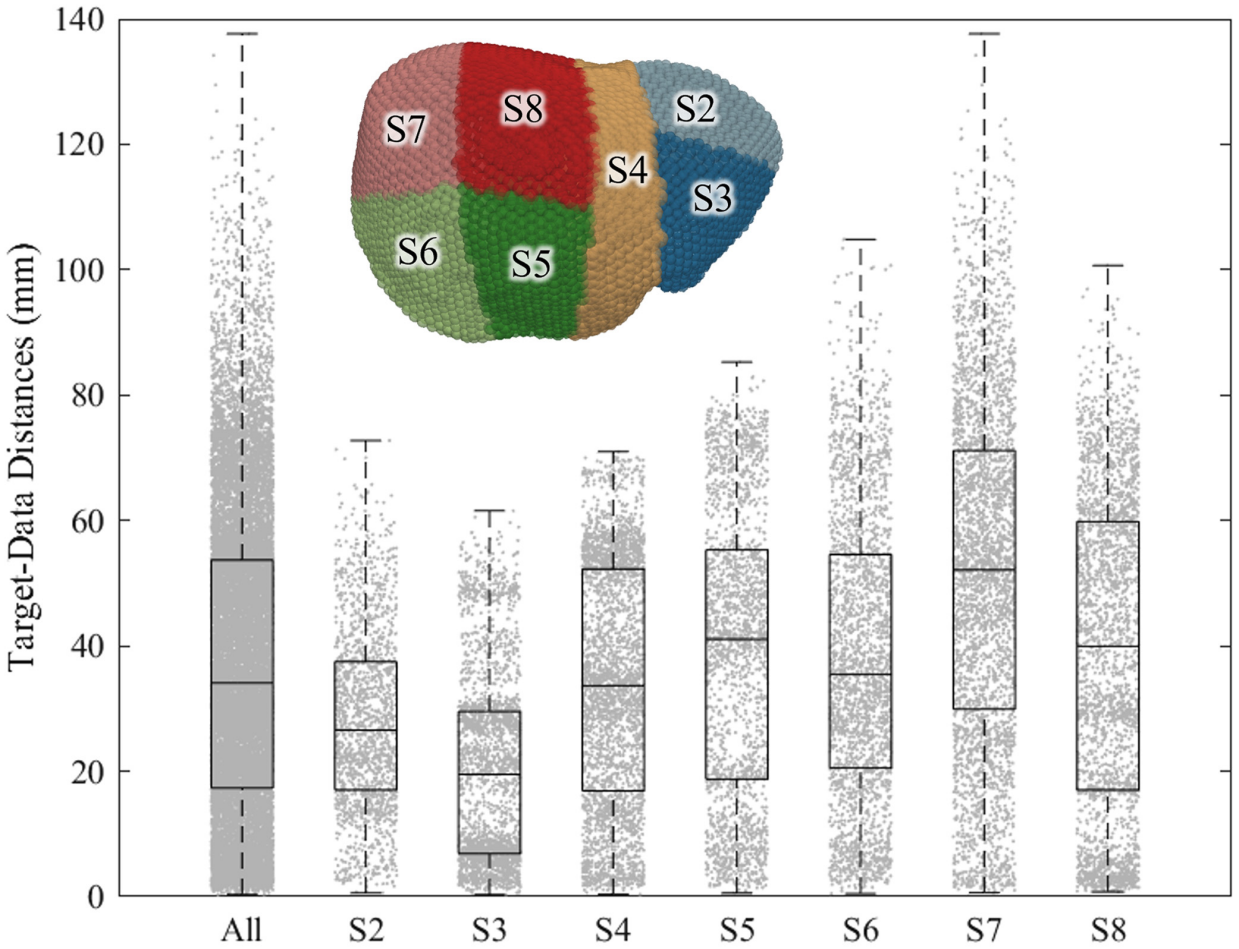
Distribution of closest-point distances from each validation target position to the intraoperative data points that are provided to drive the registration. Inset illustrates anatomical Couinaud segments marked on the sparse data challenge liver mesh.

Validation targets were partitioned into their associated Couinaud segments of the liver to evaluate variations in accuracy contingent on clinical designations of possible target locations. Figure 6 shows the segments identified on the liver mesh and the distribution of distances from each target to the nearest sparse data point in each registration scenario. Columns S2 through S8 in Table 2 summarize the TRE performances of each registration algorithm across the associated anatomical segments. Across all registration methods, TRE significantly varied depending on the anatomical location of the target (p<0.001). TRE of the deformable registration methods were found to be highest in the most peripheral segments of the liver (S2, S6, and S7) where the informational influence of sparse data tended to be weakest. Compared with S5, significantly higher TRE values were observed across registration methods in S2 (p=0.017), S6 (p=0.002), and S7 (p=0.004), whereas S6 was also found to produce significantly higher TRE than S4 (p=0.025). Intraoperatively, it should be noted that it is often not possible to collect point cloud data on the surface of S7 due to the dome of the right lobe of the liver obstructing line of sight and direct access to this area of the liver. Comparing the performance of each method across the segmental anatomy, the deep learning method of Jia and the regularized Kelvinlet method of Ringel exhibited highest TRE values in S7, which was the anatomical segment on average farthest away from the sparse data included in the dataset, whereas the deep learning method of Pfeiffer produced the least accurate inference of deformations in S2 and S6. The finite element-based biomechanical registration methods of Heiselman and Mestdagh achieved the most consistent performance across segments, although they exhibited highest errors in S6 and S7. When adjusting registration performance for the effect of target location, the only deformable registration methods to show significant improvement over ICP or wICP in all segments were those of Heiselman (p=0.044 and 0.002, respectively) and Mestdagh (p=0.063 [N.S] and 0.002, respectively), whereas all other comparisons among methods did not approach statistical significance after correction for multiple comparisons (p>0.13). These behaviors highlight the need to control uncertainties in anatomical regions that are distant from data and illuminate the benefit of biomechanical models for stabilizing performance in deformable registration algorithms. This consideration may become especially pertinent for deep learning approaches considering their tendency to develop extrapolative fragility when making inferences beyond the span of their training data.

**Table 2:** TRE performance overall and in each anatomical segment, reported as mean ± standard deviation (median).

TRE (mm)	All	S2	S3	S4	S5	S6	S7	S8
Optimal rigid registration	3.77 ± 2.40 (3.16)	5.48 ± 3.15 (4.90)	4.00 ± 1.95 (3.49)	3.79 ± 2.03 (3.35)	2.77 ± 1.58 (2.46)	5.34 ± 3.22 (4.71)	**3.14 ± 1.64 (2.69)**	**2.59 ± 1.43 (2.27)**
Manually initialized ICP	5.53 ± 3.43 (4.84)	7.71 ± 3.98 (6.76)	5.09 ± 2.30 (4.84)	5.31 ± 2.65 (5.11)	4.14 ± 2.62 (3.46)	7.43 ± 4.21 (6.36)	5.73 ± 3.62 (4.84)	3.86 ± 2.59 (3.14)
Salient feature weighted ICP (Clements)^11^	7.73 ± 4.48 (6.74)	7.65 ± 4.18 (6.53)	5.37 ± 2.64 (5.11)	5.03 ± 2.39 (4.65)	5.80 ± 2.88 (5.40)	9.15 ± 4.68 (8.47)	11.9 ± 4.54 (11.40)	8.20 ± 4.22 (7.62)
Deep learning method 1 (Pfeiffer)^12^	7.55 ± 5.28 (6.37)	10.4 ± 6.42 (8.86)	7.97 ± 4.63 (7.21)	6.46 ± 5.47 (5.32)	5.83 ± 5.02 (4.59)	9.33 ± 7.04 (7.49)	7.99 ± 9.66 (5.73)	5.79 ± 7.63 (3.99)
Deep learning method 2 (Jia)^13^	4.29 ± 3.27 (3.67)	4.63 ± 2.51 (4.16)	3.59 ± 1.74 (3.40)	3.82 ± 2.22 (3.41)	3.41 ± 2.15 (3.02)	4.77 ± 2.88 (4.13)	5.32 ± 5.08 (4.17)	4.24 ± 3.55 (3.60)
Biomechanical method 1 (Heiselman)^14^^,^^15^	**3.08 ± 1.80 (2.72)**	**3.21 ± 1.88 (2.86)**	2.62 ± 1.48 (2.36)	**2.55 ± 1.43 (2.27)**	**2.50 ± 1.28 (2.27)**	4.19 ± 2.25 (3.77)	**3.49 ± 1.78 (3.15)**	**2.98 ± 1.70 (2.61)**
Biomechanical method 2 (Mestdagh)^16^	3.31 ± 1.86 (2.95)	3.26 ± 2.11 (2.86)	**2.58 ± 1.38 (2.37)**	2.68 ± 1.45 (2.44)	2.74 ± 1.35 (2.55)	**3.85 ± 2.13 (3.43)**	4.08 ± 1.95 (3.82)	3.74 ± 1.86 (3.52)
Biomechanical method 3 (Ringel)^17^	4.61 ± 2.48 (4.18)	4.51 ± 2.52 (4.08)	3.78 ± 1.97 (3.44)	3.68 ± 1.82 (3.38)	3.48 ± 1.71 (3.22)	5.54 ± 2.87 (4.99)	6.27 ± 2.60 (6.05)	4.46 ± 2.09 (4.25)

Methods that achieve the lowest error within each segment and over all segments of the liver are emphasized in bold.

### 4.4 Sensitivity to Measurement Noise

**Fig. 7:**
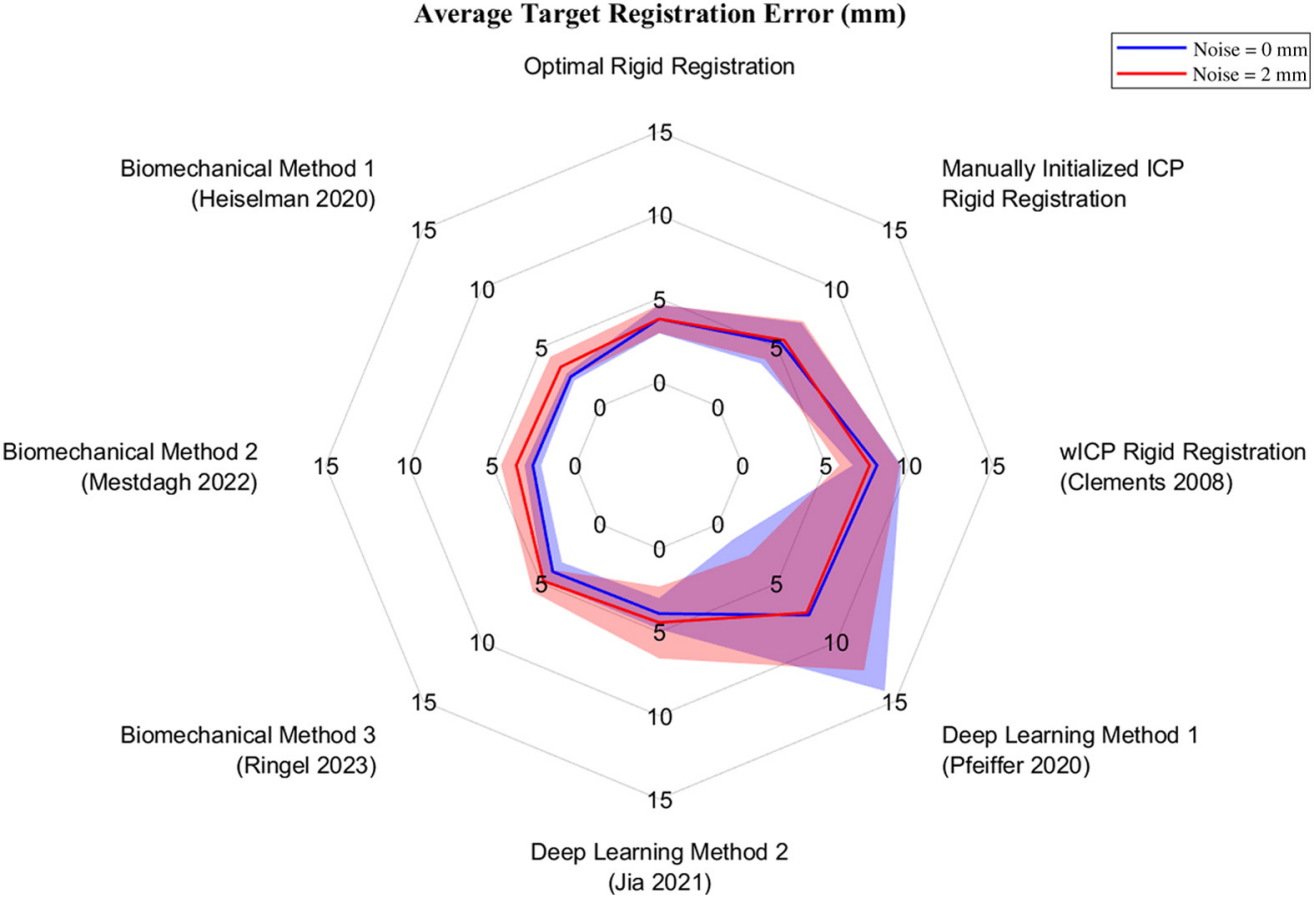
Spider plot of average TRE for noise-free registrations (blue) and registrations with added measurement noise (red). Plot shows the mean value as a solid line surrounded by a shaded region of one standard deviation.

Varying levels of measurement noise are often involved in image-to-physical registrations due to differences in data collection strategies, which may involve user variability, contact or non-contact intraoperative organ digitization techniques, or non-standardized depth reconstruction and tool localization algorithms. Measurement noise was simulated within the sparse data challenge via 84 registration datasets generated with added noise and 28 generated without noise to characterize algorithmic sensitivity to input noise sources. Figure 7 and Table 3 convey the influence of noise on average TRE of each method. The rigid registration and deep learning methods were not significantly affected by differences in input noise (all p>0.14), whereas the biomechanical methods were associated with a significant increase in mean TRE (all p<0.001). TRE variances of the finite element-based biomechanical methods also significantly increased under conditions of elevated measurement noise (largest p=0.003). Although the deep learning and rigid registration methods were less sensitive to added noise, only the biomechanical methods achieved registrations with high efficiency scores indicating TRE magnitudes on par with the relatively small noise level under investigation. Considering the modest noise magnitude, statistical analyses on the degradative effects of noise may be shrouded by the elevated level of baseline error and error variances associated with several of the other methods under investigation. Of the methods evaluated herein, only the finite element-based biomechanical boundary condition reconstruction methods were able to achieve high noise efficiency and small error variances under the 2-mm measurement noise condition.

**Table 3:** TRE performance in noise-free and noise-afflicted sparse registration datasets, reported as mean ± standard deviation.

Average TRE (mm)	Noise = 0 mm	Noise = 2 mm	Efficiency	Degradation	Average	Variance
Optimal rigid registration	3.77 ± 0.86	3.77 ± 0.86	0.56 ± 0.13	0.00 ± 0.43	p=0.91	p>0.99
Manually initialized ICP	5.34 ± 1.72	5.59 ± 1.61	0.39 ± 0.12	0.13 ± 0.82	p=0.43	p=0.91
Salient feature weighted ICP (Clements)^11^	8.06 ± 1.46	7.62 ± 1.81	0.28 ± 0.06	-0.22 ± 0.87	p=0.14	p=0.64
Deep learning method 1 (Pfeiffer)^12^	7.70 ± 6.43	7.51 ± 4.88	0.32 ± 0.11	-0.10 ± 2.65	p=0.27	p=0.61
Deep learning method 2 (Jia)^13^	3.89 ± 0.93	4.42 ± 2.15	0.51 ± 0.15	0.27 ± 0.96	p=0.16	p=0.26
Biomechanical method 1 (Heiselman)^14^^,^^15^	2.50 ± 0.31	3.34 ± 0.87	0.64 ± 0.15	0.42 ± 0.38	p<0.001	p<0.001
Biomechanical method 2 (Mestdagh)^16^	2.56 ± 0.50	3.56 ± 0.92	0.60 ± 0.16	0.50 ± 0.42	p<0.001	p=0.003
Biomechanical method 3 (Ringel)^17^	4.02 ± 0.79	4.80 ± 0.92	0.43 ± 0.08	0.39 ± 0.44	p<0.001	p=0.44

Statistically significant findings are bolded.

### 4.5 Sensitivity to Initial Alignment

**Fig. 8:**
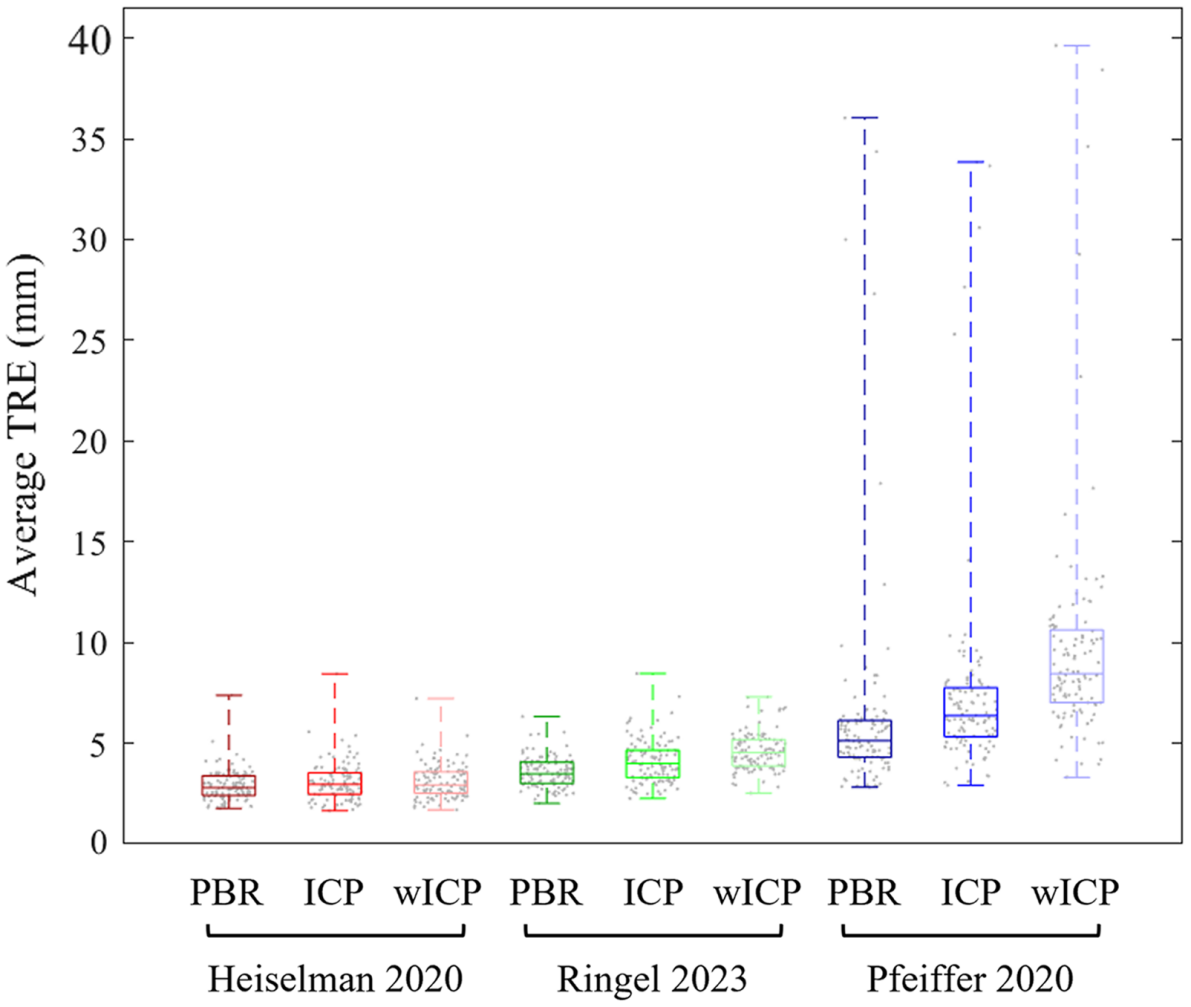
Distribution of 112 average TRE values after deformable registration starting from varying the initial pose (Optimal PBR, Manual ICP, and wICP) plotted for the biomechanical method of Heiselman (red), biomechanical method of Ringel (green), and deep learning method of Pfeiffer (blue).

All deformable registration methods with codes that were made available to the authors were further analyzed to characterize the susceptibility to differences in the choice of rigid pose that initializes the algorithm. The methods of Pfeiffer, Heiselman, and Ringel were included toward this objective. The optimal PBR, ICP, and wICP rigid alignment methods were chosen as initialization comparators for each of the three deformable registration methods, and the resulting distributions of average TRE across the 112 registrations are compared in Fig. 8. The finite element-based biomechanical strategy was robust to the initial pose, and the distributions of average TRE did not significantly shift across initialization strategies (p>0.18). However, the regularized Kelvinlet-based biomechanical strategy expressed significant differences in TRE distribution under different initial pose configurations (p<0.001), although the differences in magnitude shifted by <2  mm. The deep learning method of Pfeiffer exhibited the largest differences in average TRE when varying the initial rigid alignment strategy ($p<10^{-5}$).

### 4.6 Field Consistency

**Fig. 9:**
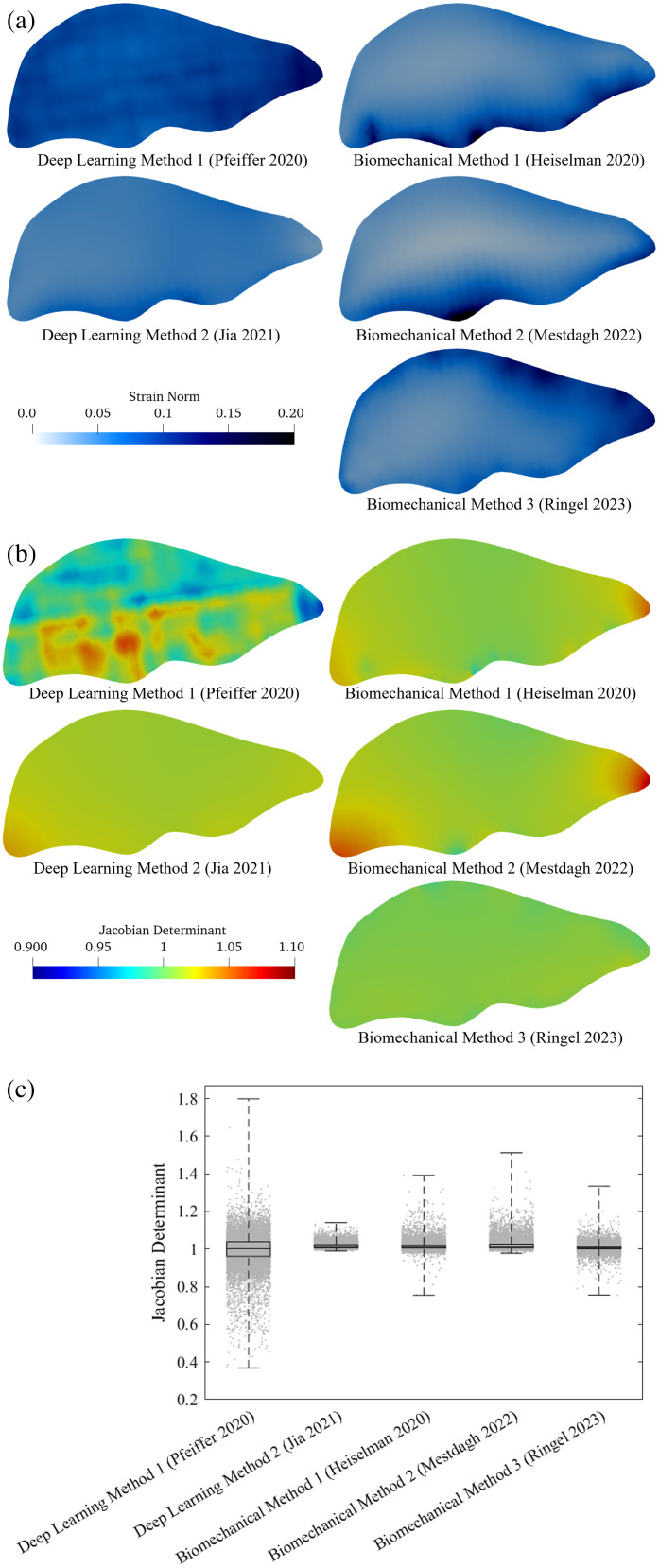
(a) Norm of the rotation-invariant Green strain tensor computed from displacement fields of the deformable registration methods. (b) Jacobian determinant of displacement fields. (c) Distribution of Jacobian determinants evaluated at all validation target locations in the set of sparse data challenge registrations.

A biomechanical analysis of displacement fields from each method was performed to analyze constitutive regularity and yield deeper insights toward current algorithmic limitations. The strain norm and Jacobian determinant of displacements on each element were averaged across the 112 displacement fields and are rendered in Fig. 9 for each deformable registration method through a cross-section of the liver. The strain norm plots indicate the locality of where each registration method expects forces to be applied over the liver, and the Jacobian determinant, which measures local volume change and is expected to equal unity for nearly incompressible soft tissue, reveals additional field inconsistencies across registration methods. Given that the underlying deformations applied to the liver phantom consisted of mock laparotomy pads placed under the posterior surface of the liver, the distribution of strain is expected to be primarily distributed along the posterior surface of the liver. Given the absence of non-gravitational constitutive body forces and free exposure of the anterior surface, the remainder of the liver in this phantom experiment is expected to associate with low strain. Briefly, the finite element-based biomechanical methods demonstrate the closest results to the expected distribution of force deposition on the liver, whereas the regularized Kelvinlet method (Ringel^15^) offers the most concordant distribution of Jacobian determinants. Overall, each method produces distinct deformation field characteristics that are impacted by several modeling decisions and algorithmic choices, which are outlined in Sec. 7 and discussed in Sec. 5.

### 4.7 Correlation Analysis

**Fig. 10:**
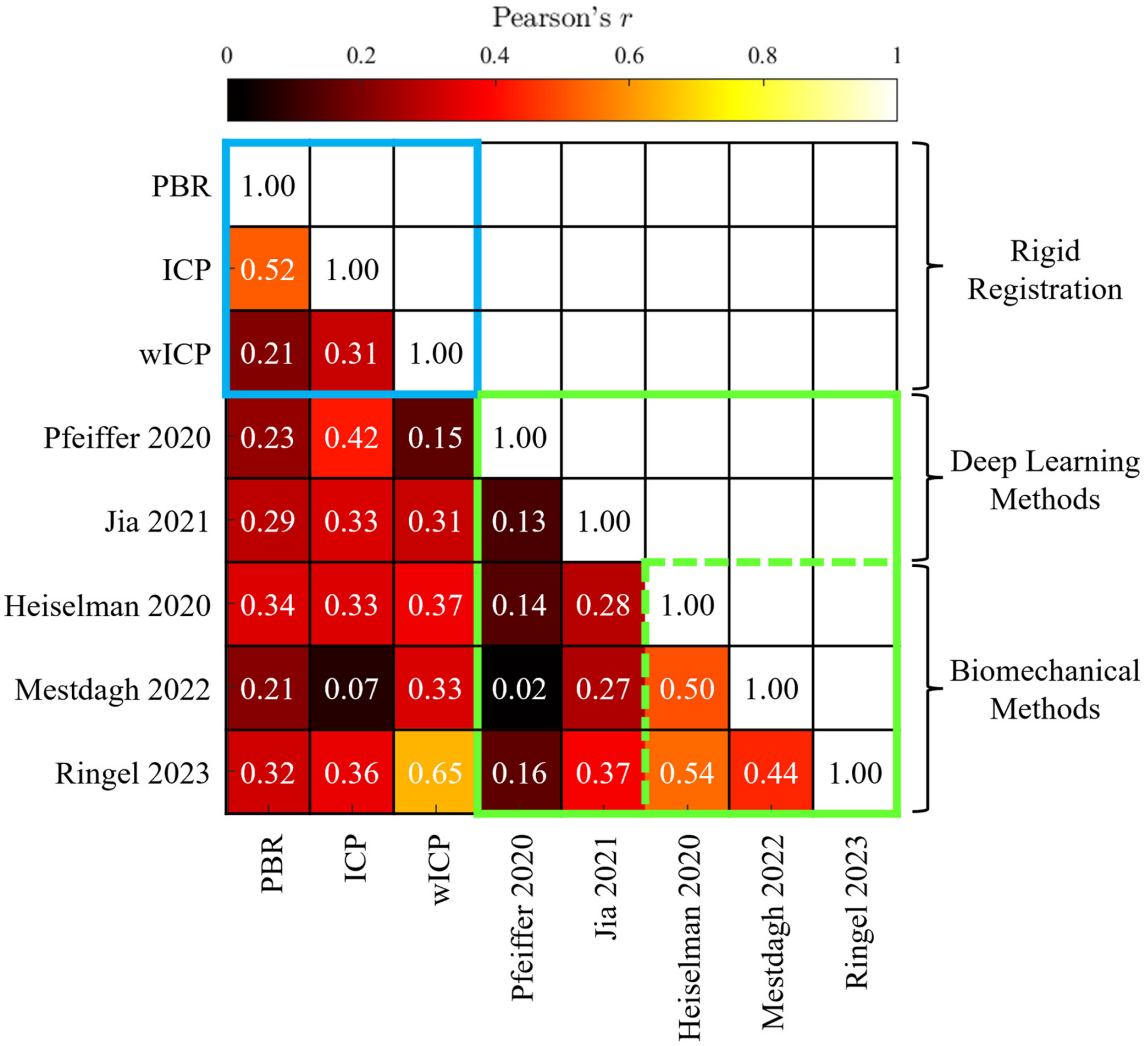
Correlation matrix of TRE among rigid registrations (blue outline) and deformable registrations (green outline). Biomechanical deformable registrations are emphasized by a dashed green outline.

Finally, Pearson correlation between individual ${\mathrm{TRE}}_{i}$ samples for each combinatorial pair of methods are plotted in Fig. 10. This correlation plot reveals that target error magnitudes within and across rigid registration and deformable registration methods are in general poorly correlated with each other, with few Pearson correlation coefficients exceeding 0.5. Notably, the deep learning method of Pfeiffer is uncorrelated with the other biomechanically informed deformable registration algorithms, with correlation coefficients below 0.16. Similarly, the deep learning method of Jia is weakly correlated, with correlation coefficients below 0.37. Across all registration methods, the strongest correlations are achieved between manually supervised ICP and optimal PBR rigid registrations, and among the three biomechanical boundary condition reconstruction algorithms. Interestingly, the regularized Kelvinlet method of Ringel showed a high correlation with the rigid wICP method of Clements that served as the initial alignment for this method, suggesting that the regularized Kelvinlet approach could be more conservative in preserving the initial pose or could exhibit stiffening effects when performing deformable registration. The overarching lack of strong correlation implies that the specific algorithm choice is profoundly important with respect to the particular way targeting inaccuracies may manifest in prospective guidance applications, since a target location predicted by one algorithm may be only weakly related or even uncorrelated with the same target location predicted by a variant algorithm. Although certain families of registration methods may exhibit greater similarity, it is therefore important for multiple registration algorithms to be compared for performance when proposing new prospective applications for image guidance under sparse data-driven deforming environments. This finding also justifies potential investigation into decision fusion methods to identify consensus registrations that combine results from multiple methods. It is interesting to note that averaging the displacement fields between the two finite element deformable registration methods improves average TRE from individual baselines of 3.08±0.85  mm and 3.31±0.94  mm for the methods of Heiselman and Mestdagh, respectively, to merely 2.93±0.75  mm across the sparse data challenge (p<0.001, paired t-test).

## 5 Discussion

Results from the sparse data challenge reveal variabilities in the effectiveness of registration strategies evaluated on a common dataset. It should be noted that all deformable registration methods utilized biomechanical deformation models to different degrees, whether through direct finite element simulation or reinforced through training in deep learning approaches. This dataset consisted of small to moderate deformation magnitudes with maximum target displacements of 11.9±3.7 (max 15.5) mm across deformation states after factoring out rigid motion, which is consistent with clinically observed deformation magnitudes for open liver surgery reported in Ref. 19. These modest deformation magnitudes suggest that the linear elastic approaches pursued by many of the participating deformable registration methods are adequately suited to the conditions associated with the sparse data challenge. Importantly, data sparsity is a considerable barrier to driving intricate models with high degrees of freedom. Biomechanical simulation offers an opportunity to incorporate underlying structure to the registration problem and restore algorithm performance under scarce informational constraints.

Target error performance of rigid registration is also revealed to be of great importance, with results showing that ICP and wICP algorithms lead to significant differences in rigid alignment and TRE. These findings suggest that determining an optimal rigid alignment from sparse surface data in the presence of underlying deformation is a non-trivial problem when the accuracy of subsurface targets is a primary concern. Due to violation of the rigid body assumption, particular attention toward the extrapolative performance of surface-based ICP registrations is needed when these approaches are applied to soft tissue organs. Moreover, Fig. 8 indicates that deformable registration algorithms may express different levels of sensitivity to variations in the initial rigid alignment. It should be noted that three of the five deformable registration methods (Heiselman,^14^^,^^15^ Jia,^13^ and Ringel^17^) take additional precautions to concurrently re-optimize rigid pose parameters during deformable registration. Considering best practices, it may be worthwhile to operate under an assumption that surface-based rigid alignments are fundamentally unreliable in the presence of soft tissue deformation. Nonetheless, the low error associated with the optimal PBR may suggest that, in some situations, a locally rigid approach may be appropriate if a sufficient number of landmarks in close proximity to a target of interest could be measured. However, accurately localized landmark data can be burdensome to collect intraoperatively. Furthermore, the findings in Table 2 suggest that the segmental location of a target of interest in the liver can profoundly affect its registration accuracy. Some biomechanical deformable registration algorithms, especially those configured to match anatomically pertinent boundary conditions, can obtain comparable or better registration errors than the most optimal point-based rigid registration while only using sparse point clouds of the intraoperative organ surface.

With respect to commonalities among methods, the two finite element-based deformable registration methods (Heiselman^14^^,^^15^ and Mestdagh^16^) made use of the fact that physical deformation was applied only to the posterior surface of the liver phantom. This *a priori* knowledge can be leveraged to reduce the complexity of the problem space and potentially improve the conditioning between latent model parameters and data constraints in optimization-based registration methods. In Heiselman and Mestdagh, this knowledge was incorporated into the registration by taking advantage of natural stress-free boundary conditions inherent to the finite element method and eliminating reconstructive degrees of freedom over the anterior surface, which is expected to remain stress-free. Notably, neither the deep learning methods nor the regularized Kelvinlet method incorporated similar *a priori* information, which is likely a distinguishing factor separating the performance of these deformable registration methods. It should be remarked that the regularized Kelvinlet method assumes the organ to be embedded in an infinite elastic medium that more naturally represents zero-displacement boundary conditions than stress-free conditions wherever degrees of freedom are removed from the reconstructive framework. Additional characterization detailed in Ref. 17 showed that the performance of the regularized Kelvinlet registration method on the sparse data challenge dataset was optimized when boundary condition reconstruction was performed over the full organ surface, unlike the behavior of finite element simulations, which benefitted from eliminating reconstructive degrees of freedom over stress-free regions. Overall, the biomechanical boundary reconstruction methods demonstrated excellent performance, and the methodologically succinct representations of anticipated boundary conditions most accurately reflecting the underlying anatomy, physiology, and clinical task seemed to offer the most success for accurately predicting motions of deforming targets from sparse intraoperative surface data. Incorporating these insights into boundary condition generation will likely remain important for training future deep learning approaches that attempt to conform to the underlying biomechanics and extrapolate soft tissue behaviors from the intraoperative locale of available data into more distant regions.

Figure 9 also illuminates how algorithmic differences and design choices may affect deviations in predicted target displacements among methods. In Fig. 9(a), the approach of Heiselman exhibits strain artifacts on the posterior surface that likely arise from the biomechanical simulation of superposed point load displacements generating numerically induced stress concentrations associated with this formulation of boundary conditions. The method by Mestdagh offers a smoother strain field, although it concentrates strain disproportionately around the portal vein entry point due to the numerical need for a fixed displacement constraint in the force-based reconstruction implemented in this approach. The deep learning method of Pfeiffer is associated with an inconsistent strain field that exhibits voxelization artifacts likely associated with data discretization procedures at input and output layers of the convolutional neural network (CNN), which is in stark contrast to the deep learning method of Jia that performs a biomechanical simulation guided by a learned point-convolutional shape occupancy objective function. Although the method of Jia produces approximately uniform strain, completely uniform strain distributions are inaccurate to the expected underlying biomechanics of elastic soft tissue deformation. The strain deposition of Heiselman and Mestdagh most closely represent the method by which the liver phantom was physically deformed with mock laparotomy pads placed under the posterior surface of the liver in an open surgery configuration. These contact forces are expected to cause the highest concentration of strain on the posterior surface of the liver. Notably, the strain deposition of the Ringel method reveals an effective force distribution applied to the anterior surface of the liver, which is less realistic to the underlying organ deformation but is likely required to compensate for the infinite elastic medium assumption of this method along a stress-free boundary.

The Jacobian determinants in Fig. 9(b) reveal that the deep learning method by Pfeiffer produces volumetric deviations of up to 10%. These large deviations likely arise due to the voxelization procedure and high training loss of this method. It should be noted that the training procedure of this method on simulated biomechanical data converged to an error of 6 mm,^12^ which likely limits the overall ability of the network to accurately represent biomechanically consistent fine structures within displacement fields. By contrast, the deep learning method by Jia displays more uniform Jacobian determinants likely attributable to the use of an underlying biomechanical model and inclusion of strain energy regularization within the objective function. In fact, strain energy regularization was utilized in each of the contributed deformable registration methods except for those of Pfeiffer and Mestdagh. Strain energy regularization will likely continue to be an effective strategy for controlling field irregularities associated with deformable registration algorithms.

In Fig. 9(b), the biomechanical registration algorithms all display approximately uniform Jacobian determinants, although the finite element-based methods tended to develop volumetric dilation within the thinner ridges of the liver. These volumetric distortions likely arise due to the use of linear elastic material simulation from a rigidly registered initial pose coordinate, wherein rotational components of finite element displacements relative to this coordinate frame will cause local dilatation due to rotation dependence of the linearized strain tensor. It should be noted that this dilatational effect can be abated by a technique used in Heiselman, Jia, and Ringel, wherein rigid transformation parameters are optimized concurrently with deformation parameters alongside strain energy regularization to redistribute local rotational effects globally throughout the mesh. It is noteworthy that the regularized Kelvinlet method of Ringel is unique among methods for its remarkable consistency with respect to the Jacobian determinant measure. This consistency is made possible due to the closed-form analytic nature of its deformation basis circumventing errors associated with numerical finite element simulation of linear elasticity. Finally, it needs to be emphasized that although the methods of Jia, Heiselman, and Mestdagh were based on numerical linear elastic simulation, the trained deformation responses in Pfeiffer instead were based on numerical simulation of a hyperelastic material. In Fig. 9(c), these choices in the underlying deformation model likely influence the relative symmetry of the Jacobian determinant around unity in Pfeiffer and Ringel as compared with the upward bias influenced by element dilatation evident in the other registration methods that employ linear elastic numerical simulation.

With respect to the behavior of TRE among methods, quantitative differences in Table 2 would suggest that the algorithmic choices discussed above have profound consequences on the final accuracy of registrations between a soft tissue organ and sparse point cloud data representing its deformed state. These differences are also reflected in the low correlation of target errors in Fig. 10 and visual differences in predicted organ shapes in Fig. 3. Registration errors may crucially depend on the locations where errors are measured in combination with the locations where forces are imparted on the organ. Consequently, intraoperative data localization, algorithm initialization, and determination of where forces may act upon the organ are remarkably important to the task of image-to-physical deformable registration. With respect to the biomechanical model-based registration algorithms, the fidelity of boundary condition composition likely played a substantial role in separating the performance of Heiselman and Mestdagh from the other deformable registration methods. Comparing against the biomechanical method of Ringel suggests that although a biomechanical deformation basis offers a useful structure for constraining the registration problem, the specific tuning of boundary condition designation for relevant anatomical and physiological factors appears to be critical for optimizing registration performance. The ability to train a generalizable expectation for task-specific anatomical motions will likely be a major next step for similarly improving the accuracy of deep learning registration algorithms driven by sparse intraoperative data.

Regarding the plateau behavior of TRE within the 20–40% extent range of data, we note in the general case that registration algorithms are often ill-posed, which results from underspecification of model parameters relative to the model constraints. Although an ordinary approach for resolving this indeterminacy would revolve around incorporating additional *a posteriori* measurement data, information located beyond the visible anterior surface would be necessary to more thoroughly inform unknown boundary conditions applied to regions where registration constraints are missing. Another potential approach to overcoming this performance plateau involves incorporating *a priori* domain knowledge such as biomechanical expectation of boundary conditions, large sets of training data, or other forms of regularization to impose bias on the registration model and its parameters. One exciting direction pursued by Jia attempts to alter the efficiency with which the data constraints inform the registration model using a learned objective function to optimize the extraction of model parameters from sparse data. However, all techniques that rely on strong *a priori* knowledge may ultimately impair generalizability. It is therefore necessary for registration algorithms to be explicitly tested for generalization performance through experimentation with unseen data or prospective validation studies that match the intended use case.

The sparse data challenge also highlights the need to design studies that consider environmental factors such as the impact of measurement noise on registration performance, which has long remained underappreciated. Although rigid registrations appear to be relatively robust to measurement noise, deformable registration methods tend to be more susceptible. Particular care should be taken during algorithmic development and evaluation to quantitate or otherwise control elevations in error magnitudes and error variances that may occur due to changes in the level of input noise. This consideration will likely become even more important when training and validating deep learning approaches within medical image registration workflows as these algorithms continue to mature.

The main limitations of the sparse data challenge include the simulation of measurement noise at only two noise amplitudes, restriction to a single baseline liver geometry, inclusion of only four distinct deformation states with modest deformations, and dependence on a synthetic silicone liver phantom over clinically obtained human validation data. In addition, the challenge does not provide subsurface data constraints to further inform registration beyond the provided sparse surface data patterns, and computational time requirements of each method were not part of the data collection process. Nonetheless, this challenge offers a detailed look into the performance of current state-of-the-art rigid and nonrigid sparse data registration algorithms for liver interventions and offers comparative insights into common and unique algorithmic traits that will continue to inform the next generation of image-to-physical sparse data registration algorithms.

## 6 Conclusion

Results were presented for the first Image-to-Physical Liver Registration Sparse Data Challenge that evaluated and compared eight distinct rigid and deformable registration approaches with respect to registration accuracy under varying conditions of data coverage, target location, and measurement noise. In addition, sensitivity to algorithm initialization, displacement field consistency, and inter-registration similarity were explored. The overarching findings showed that biomechanical deformation bases tend to achieve the best registration accuracy and field consistency among state-of-the-art methods for sparse data-driven deformable image registration. Furthermore, only the family of biomechanical boundary condition reconstruction deformable registration algorithms outperformed the best achievable rigid registration when they incorporated task-specific insight into boundary condition composition. The results of this challenge suggest that specific implementation choices profoundly affect the TRE that develop from registration algorithms and their estimated displacement fields.

## 7 Appendix A: Contributed Methods

### 7.1 Optimal Point-Based Rigid Registration

The first registration strategy is a common comparator representing an optimal PBR between the ground-truth preoperative and intraoperative positions of all 159 validation targets. A singular value decomposition approach was utilized to find the rigid transformation that minimizes the sum of squared TRE within each registration instance. Although this method incorporates unobtainable information about the true intraoperative target positions and therefore is not achievable in practice, errors associated with this globally optimal rigid registration represent a useful benchmark against which to gauge competing methods.

### 7.2 Manually Initialized Iterative Closest Point Rigid Registration

The second registration strategy is an ICP rigid registration algorithm^10^ initialized from a manually designated initial pose estimate. The ICP algorithm repeatedly updates a rigid transformation between the sparse data and their closest points on the preoperative organ model using a point-based rigid registration on each iteration. Naïve ICP algorithms are highly susceptible to local minima, and therefore the resulting rigid alignments were visually verified and re-initialized from new starting poses when any instance was deemed unsatisfactory.

### 7.3 Salient Feature Weighted Iterative Closest Point Rigid Registration (Clements)

To overcome the need for manual interaction in the rigid registration process, the third strategy evaluated a fully automatic salient feature wICP algorithm.^11^ This method biases point correspondences according to preoperatively annotated anatomical feature patches to improve intraoperative robustness of the ICP algorithm. This technique utilizes a weighted point-based registration of sparse feature points to corresponding patches on the preoperative organ surface, with weight functions controlled over an exponentially decaying iteration schedule. Although demonstrated to be more robust than naïve ICP, wICP may incorporate additional bias toward preferentially aligning the specified feature information as opposed to the overall fit of the intraoperative point cloud. The salient features included the falciform ligament and left and right inferior ridges of the liver, which are explicitly marked in the sparse data challenge intraoperative point cloud patterns in Fig. 2.

### 7.4 Deep Learning Method 1: Signed Distance Map CNN (Pfeiffer)

The fourth method is a deep learning deformable registration strategy (V2SNet^12^) based on a CNN trained on voxelized distance maps computed between a preoperative organ mesh and a partial data patch of the deformed intraoperative organ surface. Briefly, after an initial rigid registration is applied to align intraoperative data with the mesh, the network estimates a function $F({\mathrm{SDF}}_{P},{\mathrm{DF}}_{I})∼u$, where ${\mathrm{SDF}}_{P}$ is the voxelized signed distance map of the preoperative organ mesh, ${\mathrm{DF}}_{I}$ is the voxelized unsigned distance map of the partial intraoperative surface to the preoperative mesh, and u is the estimated displacement field of the deformation. Training data for this method were generated from random organ mesh shapes on which random deformations with known ground truth were simulated using a hyperelastic biomechanical model. The neural network was trained in a multiresolution supervised manner according to a mean absolute error loss function:

$$L=\underset{r\in \{8,16,32,64\}}{\sum }\frac{w_{r}}{r^{3}}\overset{r^{3}}{\underset{i=1}{\sum }}|u_{r,GT}(i)-u_{r,\mathrm{est}}(i)|,$$ (6)

where $u_{r,\mathrm{GT}}$ is the ground truth voxel displacement; $u_{r,\mathrm{est}}$ is the estimated voxel displacement; r is the resolution of the voxelized image; and $w_{r}$ is a resolution-dependent training weight hyperparameter. The authors report that training data converged to mean error of ∼6  mm.^12^ Pretrained network weights at the maximum resolution were used without retraining for inference of the liver model and intraoperative data associated with the sparse data challenge, using initial ICP rigid registration alignments from Sec. 7.2.

### 7.5 Deep Learning Method 2: Probabilistic Occupancy Map PCNN (Jia)

The fifth registration strategy is a data-driven nonrigid approach^13^ based on a learned occupancy map and point convolutional neural network (PCNN) to predict the likelihood that a particular volumetric shape takes a certain configuration based on a sparse input point cloud. The authors propose a deep neural network to model a differentiable occupancy map $g(x_{i},P)\in [0,1]:\;R^{3}\to R$ for the probability that a shape occupies position $x_{i}$ given a point cloud P that describes a sparse representation of the shape surface. The occupancy map g then represents a fuzzy boundary of the liver over the spatial support of x, and an isocontour of g represents an estimated organ shape. Agreement between the occupancy of a deforming preoperative liver model and a rigidly registered intraoperative point cloud is then optimized alongside a strain energy penalty to determine a set of rigid and nonrigid deformation parameters {ϕ,t,θ} according to the following objective function:

$$C(\phi ,t,\theta )=\frac{1}{N}\overset{N}{\underset{i=1}{\sum }}{\left[g(f(x_{i}|\phi ,t,\theta ),P)-0.5\right]}^{2}+\alpha E(\phi ),$$ (7)

where $f(x_{i}|\phi ,t,\theta )$ is a transformation function that applies a deformation field parameterized by ϕ and rigid translations and rotations t and θ. In this work, the authors define $f(x_{i}|\phi ,t,\theta )$ according to a superposed finite element deformation basis introduced in Ref. 3. The penalty term αE(ϕ) represents regularization by the strain energy E(ϕ) in the manner of Rucker et al.^20^ More information and details about this method are provided in Ref. 13. Unlike the end-to-end deep learning method in Sec. 7.4, which attempts to learn a deep deformation basis to alleviate the need for intraoperative biomechanical simulation, this deep learning approach incorporates biomechanical simulation to establish a network-based filter for model-data correspondence errors. Compared with conventional simulation approaches that assume a model-data correspondence function and minimize the error between the set of observed data points and their corresponding locations on the deforming organ model, this technique explores an interesting framework for learned objective functions that alternatively encode preoperative-to-intraoperative correspondences through probabilistic model occupancy, which may offer new approaches to offset deleterious effects of measurement noise and uncertainty associated with anatomical correspondences in image-to-physical registration to sparse point cloud data. A unique feature of the PCNN model is that data voxelization is not required because the occupancy function can be sampled continuously through space at any point of interest.

### 7.6 Biomechanical Method 1: Linearized Iterative Boundary Reconstruction (Heiselman)

The sixth registration strategy is a linearized iterative boundary reconstruction method^14^^,^^15^ that uses a biomechanical finite element model of the organ to control nonrigid deformation. The method reconstructs a set of boundary conditions applied to a mesh of the preoperative organ that best explains the intraoperative deformation state observed in sparse data measurements of the organ surface. This technique decomposes the mechanical load applied to the boundary of the organ into a set of localized point forces distributed over the active contact surfaces of the organ. These local point forces are superposed to allow for rapid estimation of the deformation state from a precomputed basis of perturbation responses obtained from a linear elastic finite element model. An intraoperative deformation state is obtained by iteratively optimizing the following weighted least squares objective function:

$$C(\alpha ,t,\theta )=\underset{F}{\sum }\frac{w_{F}}{N_{F}}\overset{N_{F}}{\underset{i=1}{\sum }}f_{i}^{2}+w_{E}f_{E}^{2}$$ (8)

where $f_{i}=f_{i}(\alpha ,t,\theta )$ is the Euclidean model-data error associated with the sparse data point i in feature F and the penalty term $f_{E}=f_{E}(\alpha )$ is the strain energy of the deformation state for the vector of deformation basis weights α. The relative contributions of error terms and consequently the deformability of the registration is controlled by the ratio of the weight factors $w_{F}$ and $w_{E}$. A total of 20 control points were distributed across the posterior surface of the liver to match the loading configuration applied to the organ in this dataset. The method is initialized with rigid transformation parameters determined by the wICP algorithm in Sec. 7.3 to establish an automatic and robust starting alignment after which the rigid and nonrigid parameters are simultaneously optimized in a Levenberg–Marquardt framework.

### 7.7 Biomechanical Method 2: Adjoint Boundary Reconstruction (Mestdagh)

The seventh registration strategy is a biomechanical method using a linear elastic finite element model within an adjoint optimization scheme that solves for boundary forces applied to the posterior surface of the liver mesh.^16^ First, an ICP rigid registration algorithm is applied prior to initiating the deformable registration. Then, an adjoint method is developed to iteratively minimize the least squares objective function:

$$C(b)=\frac{1}{2N}\overset{N}{\underset{i=1}{\sum }}{\left\|p(y_{i}|u_{b})-y_{i}\right\|}^{2},$$ (9)

for nodal boundary forces b with associated displacement field $u_{b}$ and adjoint state $p(y_{i}|u_{b})$ for an observed sparse data point $y_{i}$. To numerically solve the system of equations, displacements over a small patch on the posterior face of the liver near the portal vein insertion point are fixed to zero-displacement Dirichlet boundary conditions. In contrast to the displacement-driven linearized method in Sec. 7.6, this method utilizes a force-based reconstruction over an iteratively updating forward model solution process. This method may be extended to hyperelastic and other nonlinear deformation models, although more sophisticated deformation models may incur potentially prohibitive costs to computation time. Notably, due to the adjoint approach, this linear elastic model does not concurrently optimize rigid transformation parameters.

### 7.8 Biomechanical Method 3: Regularized Kelvinlet Boundary Reconstruction (Ringel)

The eighth registration strategy builds upon the linearized iterative boundary reconstruction approach of Sec. 7.6 and similarly decomposes the mechanical load applied to the organ into a series of localized control point responses. However, this method replaces the finite element simulations with closed-form displacement equations associated with Kelvin state solutions established in formal elastic theory. The Kelvin state analytically models the linear elastic displacement response of a point load perturbation embedded within an infinite linear elastic domain. These point load responses can be superposed to establish a deformation basis consisting of a series of spatially localized Kelvinlet deformations that are distributed across the boundary of the organ. A regularization approach is incorporated to extend the analytic Kelvin response from a point load impulse to a spatially localized smoothed force density function. The regularized Kelvinlet displacement solutions are analytic algebraic equations that remove the need for computationally expensive finite element simulation and greatly accelerate realistic biomechanical simulation by leveraging classical solution methods to 3D linear elasticity. The regularized Kelvinlet displacement solution $u_{\varepsilon }$ to a local force perturbation is defined as

$$u_{\varepsilon }(r)=(\frac{a-b}{r_{\varepsilon }}I+\frac{b}{r_{\varepsilon }^{3}}rr^{T}+\frac{a\varepsilon^{2}}{2r_{\varepsilon }^{3}}I)f_{0},$$ (10)

where $f_{0}$ is the force magnitude applied to the Kelvinlet center point, r is the radial distance from the Kelvinlet center to any position in the 3D spatial support, $r_{\varepsilon }=\sqrt{r^{2}+\varepsilon^{2}}$ is a regularized radius incorporating a radial scale ε, and a and b are material constants that depend on elastic modulus and compressibility. The regularized Kelvinlet displacement basis is used in the same reconstructive framework as the method of Ref. 14 in Sec. 7.6 with an identical objective function to Eq. (8), except a larger number of 160 control points distributed over the complete liver surface are required to reach optimal algorithmic performance. Additional characterization and implementation details are provided in Ref. 17. It should be noted that, compared with the finite element simulation, this method assumes a specific local force density function that depends on the regularization parameter ε, and its assumption of an infinitely homogeneous elastic medium renders the solution less adaptive to patient-specific organ geometry and mechanically disjoint contact interfaces.

## Data Availability

The complete sparse data challenge dataset including unblinded target validation data and analysis techniques are available for download via the Open Science Framework at Ref. 8.
